# Molecular survey of coccidian infections of the side-blotched lizard *Uta stansburiana* on San Benito Oeste Island, Mexico

**DOI:** 10.1051/parasite/2018043

**Published:** 2018-08-15

**Authors:** Petra Quillfeldt, Tanja Romeike, Juan F. Masello, Gerald Reiner, Hermann Willems, Yuliana Bedolla-Guzmán

**Affiliations:** 1 Department of Animal Ecology & Systematics, Justus Liebig University Giessen Heinrich-Buff-Ring 26-32 35392 Giessen Germany; 2 Department of Clinical Veterinary Sciences, Justus Liebig University Giessen Frankfurter Str. 112 35392 Giessen Germany; 3 Grupo de Ecología y Conservación de Islas, A.C. Moctezuma 836, Zona Centro 22800 Ensenada Baja California Mexico

**Keywords:** Blood parasites, Lizard, *Lankesterella*, Coccidians, Eimeriorina, *Uta stansburiana*

## Abstract

Blood parasites are found in many vertebrates, but the research on blood parasites of lizards is still at its onset. We analyzed blood samples from side-blotched lizards *Uta stansburiana* from San Benito Oeste Island, Mexico, to test for the presence of hemoparasites. We found a high prevalence (23 out of 27 samples) of a blood parasite of the genus *Lankesterella* (Coccidia, Eimeriorina, Lankesterellidae) according to phylogenetic analyses of the parasite 18S rRNA gene. Similar parasites (97–99% similarity) have recently been described for *Uta stansburiana* from California. The parasite 18S rRNA gene showed high variability, both within San Benito and compared to California. The next closest matches of the parasite DNA with 97–98% similarity included a range of different genera (*Lankesterella*, *Schellackia*, *Eimeria*, *Isospora* and *Caryospora*). A high uncertainty in the deeper branches of the phylogenetic trees, and many missing links in genetic network analysis, were in line with previous suggestions that the coccidians are an understudied group with large knowledge gaps in terms of their diversity and taxonomy. Further studies are needed to resolve the evolutionary relationships within the Eimeriorina.

## Abstract (fr)

On trouve des parasites sanguins chez de nombreux vertébrés, mais la recherche sur les parasites sanguins des lézards est encore à ses débuts. Nous avons analysé des échantillons de sang de lézards, *Uta stansburiana*, de l’île de San Benito Oeste, au Mexique, pour tester la présence d’hémoparasites. Nous avons trouvé une prévalence élevée (23 échantillons sur 27) d’un parasite sanguin du genre *Lankesterella* (Coccidia, Eimeriorina, Lankesterellidae) selon les analyses phylogénétiques du gène de l’ARNr 18S du parasite. Des parasites similaires (97–99 % de similarité) ont été récemment décrits chez *Uta stansburiana* de Californie. Le gène de l’ARNr 18S du parasite présentait une variabilité élevée, tant à San Benito qu’en Californie. Les appariements les plus proches de l’ADN du parasite avec une similitude de 97–98 % comprenaient une gamme de genres différents (*Lankesterella*, *Schellackia*, *Eimeria*, *Isospora* et *Caryospora*). Une forte incertitude dans les branches les plus profondes des arbres phylogénétiques, et de nombreux chaînons manquants dans l’analyse des réseaux génétiques étaient en accord avec les suggestions précédentes selon lesquelles les coccidiens sont un groupe peu étudié avec de grandes lacunes en termes de connaissance de leur diversité et leur taxonomie. D’autres études sont nécessaires pour résoudre les relations évolutives au sein des Eimeriorina.

## Introduction

Parasites play an important role in evolution. As they constantly co-evolve with their host, parasites have direct impacts on natural communities and are a key factor in ecosystems [11]. Parasitic infections can have a negative impact on the body condition of their hosts and lead to reduced expression of sexual ornaments [32] and decreased reproductive success [16, 38, 51].

The phylum Apicomplexa contains a large diversity of single-celled eukaryotic organisms, known to parasitize vertebrates, including humans, and invertebrate hosts. The Apicomplexa are a poorly studied group, where 1.2–10 million species have been estimated, but only about 0.1% have been named and described to date [2, 37]. A review concluded that much more widespread sampling needs to occur before any reliable phylogenies are likely to emerge [36]. Within the more than 300 recognized genera of Apicomplexa, there is a major bias in knowledge towards just five genera: *Babesia*, *Cryptosporidium*, *Plasmodium*, *Theileria* and *Toxoplasma* account for 98% of the nucleotide sequences of Apicomplexa found in the GenBank database [36]. Thus, although Apicomplexans constitute one of the largest components of world biodiversity, they are possibly the most poorly known large taxonomic group, in terms of biodiversity [2], although environmental genomic information has recently resulted in a wealth of new information (e.g. [3]). Taxon and character sampling still seem to be the most serious impediments to elucidating apicomplexan phylogeny.

Of the protozoan blood parasites of vertebrates, the best studied group is the suborder Haemosporina (e.g. genera *Plasmodium*, *Haemoproteus*, *Leucocytozoon*), which is common in birds and mammals. A specific database for these parasite genera in birds has been set up [6]. However, much less is known of the eight genera of the suborder Adeleorina, and the two genera (*Lankesterella* and *Schellackia*) of the suborder Eimeriorina. Many hemogregarines have been described in snakes and lizards [43].

In the present study, we analyzed blood samples collected from side-blotched lizards *Uta stansburiana* from San Benito Oeste Island, off the Mexican Pacific coast. Previous studies have suggested infection of *Uta stansburiana hesperis* in Santa Cruz Island, Southern California with *Schellackia occidentalis* [8]. A recent study of *Uta stansburiana hesperis* from Corn Springs (southern California) and from Los Baños (western California) confirmed the presence of *S. occidentalis* through microscopic examination of blood smears [31]. However, the phylogenetic analyses indicated that the 18S rRNA sequences were distant from *Schellackia* species found in Old World lizards, but were closely related to the genus *Lankesterella* Labbé, 1899. The suggested new nomenclature for this parasite is *Lankesterella occidentalis* (Bonorris & Ball, 1955) [31].

We therefore tested if this parasite also infects side-blotched lizards from the San Benito Islands, and if so, determined whether the geographic isolation led to genetic differences.

## Materials and methods

### Study site and study species

The study took place on San Benito Oeste Island, the largest of a group of three small islands off the Pacific coast of Baja California, Mexico (28°18′N, 115°35′W). Side-blotched lizards *U. stansburiana stellata* are very common on this island. They belong to the family Phrynosomatidae (Squamata) and are small iguanid lizards living on the Pacific coast of North America with body lengths up to 7 cm. Side-blotched lizards are generalists, but their main diet consists of small arthropods and they reproduce all year long [4]. Populations from San Benito and nearby Cedros Island were formerly separated as distinct species *Uta stellata* and *U. concinna*, but are now included in *U. stansburiana*.

### Field work

Field work took place from August to September 2014. Lizards were caught by hand or in traps made from empty 5-L plastic water bottles with tomato juice and pieces of fresh or dried fruit used as bait. The base of the tail was disinfected with ethanol. Blood samples (*n* = 27) were drawn with a sterile syringe (0.33 mm, 29G) from the caudal (tail) vein, and a drop was transferred onto a Whatman FTA classic card. After sample collection, the lizards were released at the capture site.

### Laboratory analyses

In the laboratory, a 2×2 mm piece of the dried blood sample was cut out of the FTA card and the DNA was isolated using an ammonium acetate protocol (adapted from [26]). The final DNA concentration of the sample was determined with a NanoDrop2000c UV-Vis spectrophotometer (NanoDrop Technologies, Wilmington, DE, USA).

DNA samples were screened for the presence of parasitic DNA by PCR using primers HepF300 (5′-GTTTCTGACCTATCAGCTTTCGACG-3′) and Hep900 (5′-CAAATCTAAGAATTTCACCTCTGAC-3′) that target a part of the 18S rDNA gene in *Hepatozoon spp*. [47]. These primers were designed to amplify 633 bps of *Hepatozoon* DNA, but are also found to amplify DNA of other parasite species like *Eimeria* and *Sarcocystis* [17]. To obtain longer sequences for Sanger sequencing, we designed a new primer set Hep600F1N (5′-CTCGTAGTTGGATTTCTGTCG-3′) and Hep1615R (5′-AAAGGGCAGGGACGTAATC-3′, [27]), which amplifies 1029 bps of the DNA sequences (18SrRNA gene).

PCR amplicons were separated by gel electrophoresis in a 1.5% agarose gel stained with Midori Green^TM^ (Biozym, Hessisch Oldendorf, Germany) and 1× TAE (40 mM Tris, 20 mM acetic acid, 1 mM EDTA) as an electrophoresis buffer. A negative and a positive control (sample DNA of a Least storm-petrel (*Oceanodroma microsoma*) infected with *Hepatozoon peircei* [34]) were also included.

PCR reactions were run in a total volume of 16 μL containing 20 ng of template DNA, 8 μL Multiplex mastermix (Qiagen, Hilden, Germany) with 3 mM MgCl_2_ and 0.2 μM of each primer. Reactions were cycled at the following parameters using a Biometra TPersonal Thermocycler (Biometra, Göttingen, Germany): 94 °C for 15 min (polymerase activation), nine cycles at 94 °C for 30 s, 65 °C for 90 s (annealing temperature was reduced by 1 °C each step), and 72 °C for 30 s. Finally, 30 cycles were performed at 95 °C for 30 s, 55 °C for 90 s and 72 °C for 30 s, and a final extension at 72 °C for 10 min.

PCR amplicons were visualized on agarose gels under UV light. PCR products from the samples with the strongest bands on the gel were sent to the Konrad Lorenz Institute of Ethology (Vienna, Austria) for sequencing with an Applied Biosystems 3130xl Genetic Analyzer (Life Technologies, Carlsbad, CA, USA). Forward and reverse sequences were aligned using Codon Code Aligner 5.0 (CodonCode Corporation, Centerville, MA, USA) and sequences with any ambiguous positions were excluded. Sequences were aligned to sequences deposited in the GenBank nucleotide database using Blast (https://blast.ncbi.nlm.nih.gov). Related sequences with the highest similarity (97–99%) were downloaded. The downloaded sequences, the consensus sequences from our own samples, and a sequence from *Toxoplasma gondii* (GenBank accession no. EF472967) as the outgroup were aligned in BIOEDIT [15] using the ClustalW multiple alignment tool. The final length was 1279 nucleotides, which contained 333 polymorphic sites, of which 147 were parsimony-informative.

A phylogenetic tree was inferred with the reference sequences and outgroup. The final alignment included 48 nucleotide sequences (one outgroup, 19 sequences from this study and 28 reference sequences).

The best suitable nucleotide substitution model (TN93 [44], with gamma distribution and invariant sites) for our alignment was determined jModelTest 2.1.7 [10] using Bayesian Information Criterion scores. A Bayesian phylogenetic tree was generated with BEAST v1.8.4 [12]. Model parameters for this analysis were selected in BEAUTi v1.8.4 with the TN93+I+G substitution model, strict clock as clock type and a Yule speciation process [14] as tree prior. The chain length for the Metropolis coupled Markov Chain (MCMC) was set to 25 Mio. generations (burn-in 10%), and one tree was recorded every 1000 generations. Using Tracer v1.6 [40], we verified the trace for convergence. We used TreeAnnotator in BEAST v1.8.4 to generate a maximum clade credibility tree (MCCT). Finally, FigTree v1.4.3 [39] was used to visualize the final phylogenetic tree. Similarities between sample sequences were calculated in BLAST (https://blast.ncbi.nlm.nih.gov/Blast.cgi). A Maximum Likelihood analysis was also performed for comparison, using the same dataset and nucleotide substitution model (Supplement 1).

A median-joining haplotype network was estimated using PopART (http://popart.otago.ac.nz). The 19 sequences were deposited in GenBank with accession numbers MH459280–MH459298.

For the morphological inspection of blood parasites, blood smears were stained with Giemsa stain and a monolayer of blood cells was scanned with a light microscope (1000×, oil immersion, Bresser Researcher Trino 40×–1000×, with Amscope MU300 3 MP microscope camera) for parasites. Sample vouchers (blood smears) will be deposited in a curated collection (Naturkundemuseum Stuttgart, Germany).

## Results

**Figure 1.**
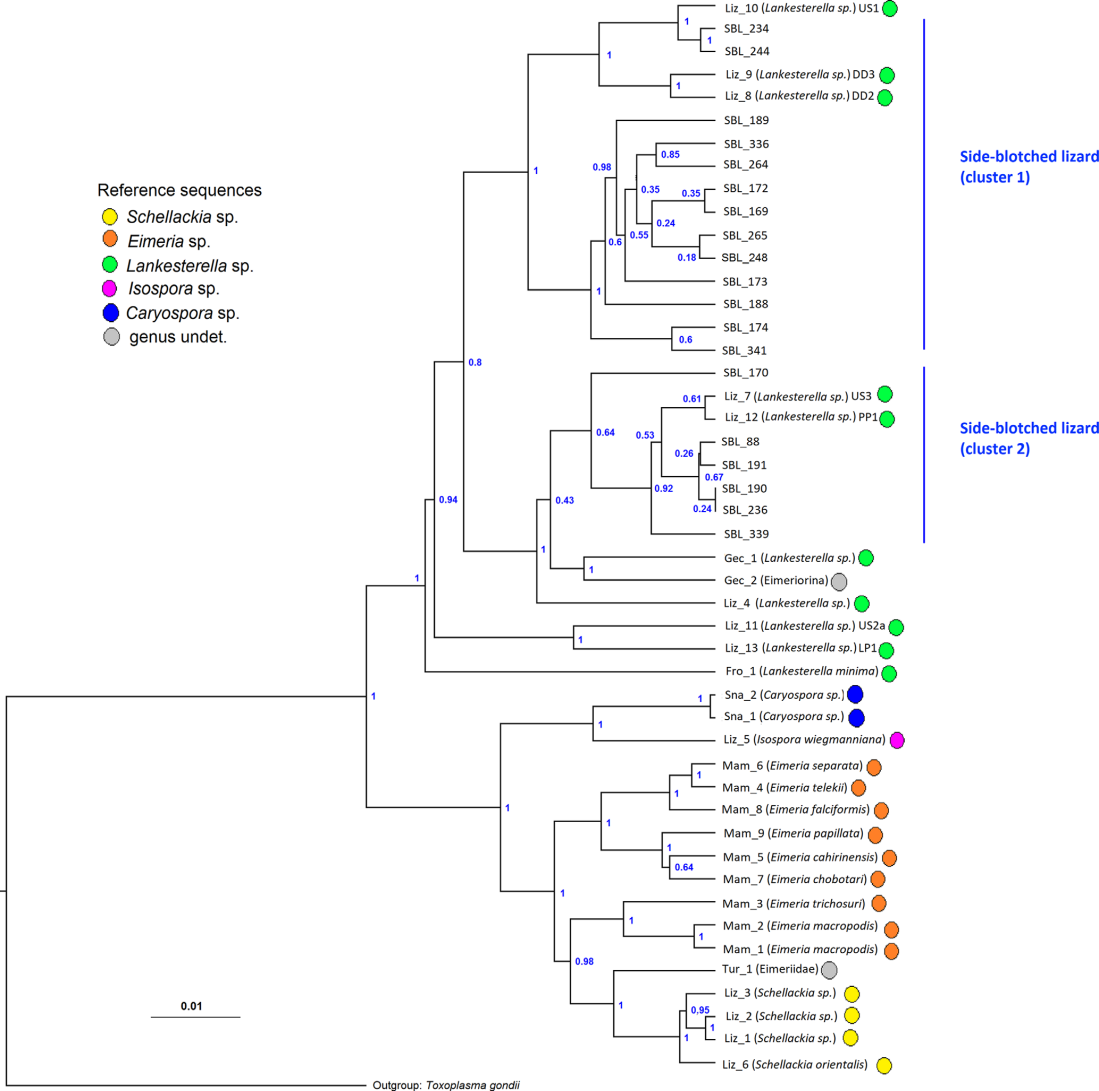
Molecular Phylogenetic analysis by Maximum Likelihood Bayesian Analysis, based on parasite DNA sequences (18S rRNA gene). Phylogenetic relationships of blood parasites found in side-blotched Lizards (SBL) *Uta stansburiana* on San Benito Oeste Island. Posterior probabilities of the nodes are shown. Details of the reference sequences, including blood parasite and host species identity and code and location, are given in Table 1. The tree is drawn to scale, with branch lengths measured in the number of substitutions per site. The analysis involved 48 nucleotide sequences (one outgroup, 19 sequences from this study and 28 reference sequences).

A total of 23 of 27 samples (85%) were PCR-positive. We successfully sequenced 19 PCR products. The Blast search revealed a 97–99% identity with published sequences from a variety of *Eimeriorina* (Table 1). We found highly similar (99%) sequences for all samples except one (SBL_170, highest similarity 97%). In the Bayesian phylogenetic tree (Figure 1), the sequences from the present study formed two clusters. Thirteen samples (68%) formed a unique cluster together with *Lankesterella* sp. haplotypes US1, DD2 and DD3 with 100% support (cluster 1 in Figure 1). The remaining six sequences formed a separate unique cluster, albeit with weaker support (95%, cluster 2 in Figure 1, related to *Lankesterella* sp. haplotype US3 found in side-blotched lizards in California, as well as a *Lankesterella* haplotype found in lizards *Phymaturus payuniae* in Argentina (PP1, Figure 1).

**Table 1.** Reference sequences for the phylogenetic relationships in Figure 2 obtained from GenBank.

CODE	Blood parasite (haplotype)	GenBank accession number	Host species	Location	Reference
Lizards					
Liz_1	*Schellackia sp.*	JX984676	*Podarcis guadarramae*	Spain	[29]
Liz_2	*Schellackia sp.*	JX984675	*Lacerta schreiberi*	Spain	[29]
Liz_3	*Schellackia sp.*	JX984674	*Lacerta schreiberi*	Spain	[29]
Liz_4	*Lankesterella sp.*	KU180248	*Anolis carolinensis*	Spain: pet trade	[31]
Liz_5	*Isospora wiegmanniana*	KU180242	*Trogonophis wiegmanni*	Chafarinas Islands	[31]
Liz_6	*Schellackia orientalis*	KC788221	*Takydromus sexlineatus*	Spain: pet trade. Origin: Thailand.	[30]
Liz_7	*Lankesterella sp.*(US3)	MF167544	*Uta stansburiana*	USA	[32]
Liz_8	*Lankesterella sp.*(DD2)	MF167545	*Dipsosaurus dorsalis*	USA	[32]
Liz_9	*Lankesterella sp.*(DD3)	MF167546	*Dipsosaurus dorsalis*	USA	[32]
Liz_10	*Lankesterella sp.*(US1)	MF167549	*Uta stansburiana*	USA	[32]
Liz_11	*Lankesterella sp.*(US2a)	MF167552	*Uta stansburiana*	USA	[32]
Liz_12	*Lankesterella sp.*(PP1)	MF167554	*Phymaturus payuniae*	Argentina	[32]
Liz_13	*Lankesterella sp.*(LP1)	MF167555	*Liolaemus pictus*	Chile	[32]
Frogs					
Fro_1	*Lankesterella minima*	KT184358	*Lithobates clamitans*	Canada	[38]
Geckos					
Gec_1	*Lankesterella sp.*	KX453658	*Hemidactylus hajarensis*	Oman	[26]
Gec_2	Eimeriorina (genus undet.)	KM234611	*Hemidactylus agrius*	Brazil	[18]
Snakes					
Sna_1	*Caryospora sp.*	KT184331	*Sistrurus catenatus*	Canada	[38]
Sna_2	*Caryospora sp.*	KT184332	*Sistrurus catenatus*	Canada	[38]
Turtles					
Tur_1	Eimeriidae (genus undet.)	KT956976	*Dermochelys coriacea*	USA	[13]
Mammals					
Mam_1	*Eimeria macropodis*	JQ392575	*Macropus eugenii*	Australia	[19]
Mam_2	*Eimeria macropodis*	JQ392576	*Macropus eugenii*	Australia	[19]
Mam_3	*Eimeria trichosuri*	FJ829322	*Trichosurus cunninghami*	Australia	[38]
Mam_4	*Eimeria telekii*	AF246717	*Lemniscomys striatus*	Czech Republic	[36]
Mam_5	*Eimeria cahirinensis*	JQ993645	*Acomys dimidiatus*	Czech Republic	[23]
Mam_6	*Eimeria separata*	AF311643	Rodents	unknown	[50]
Mam_7	*Eimeria chobotari*	AF324214	Rodents	unknown	[50]
Mam_8	*Eimeria falciformis*	KT184339	Mus musculus	USA	[38]
Mam_9	*Eimeria papillata*	KT184350	Mus musculus	USA	[38]

The mean similarity of the 18 sequences of SBL samples was 97% (range 89–100%, Table 2). Within clades (Figure 1) high similarities were observed (cluster 1: mean 99%, range 96–100%, cluster 2: mean 90%, range 94–100%), while lower similarities were found when comparing sequences of cluster 1 to those of cluster 2 (mean 95%, range 89–97%).

**Table 2.** Pairwise similarities between sequences of coccidian blood parasites found in side-blotched lizards (SBL) *Uta stansburiana* on San Benito Oeste Island.

Sample		169	170	172	173	174	188	189	190	191	234	236	244	248	264	265	336	339	341
	Cluster	1	2	1	1	1	1	1	2	2	1	2	1	1	1	1	1	3	1
88	2	97	98	97	97	97	97	97	100	100	93	100	93	97	97	97	97	99	97
169	1		96	99	99	99	99	98	97	96	97	95	97	100	99	99	99	89	99
170	2			96	96	96	96	96	98	97	92	97	92	96	96	96	96	94	96
172	1				99	99	99	99	97	96	99	96	100	100	99	99	99	91	99
173	1					99	99	99	97	96	98	96	99	100	99	99	99	91	99
174	1						99	99	97	97	98	95	99	99	99	98	99	91	99
188	1							99	97	96	97	96	98	99	99	99	99	92	99
189	1								97	97	98	97	98	100	98	99	98	91	99
190	2									100	93	100	94	97	97	97	97	99	97
191	2										91	99	91	97	96	97	96	99	97
234	1											91	100	99	96	98	98	96	99
236	1												91	97	96	96	95	96	97
244	1													100	99	96	98	96	99
248	1														99	100	100	93	99
264	1															98	99	91	99
265	1																99	90	99
336	1																	92	99
339	3																		92

A variety of *Eimeriorina* (genera *Eimeria*, *Isospora*, *Caryospora* and *Schellackia*) from lizards and a diverse range of hosts including snakes, geckos and mammals, were also related to the *Lankesterella* sp. haplotypes (Figure 1).

**Figure 2.**
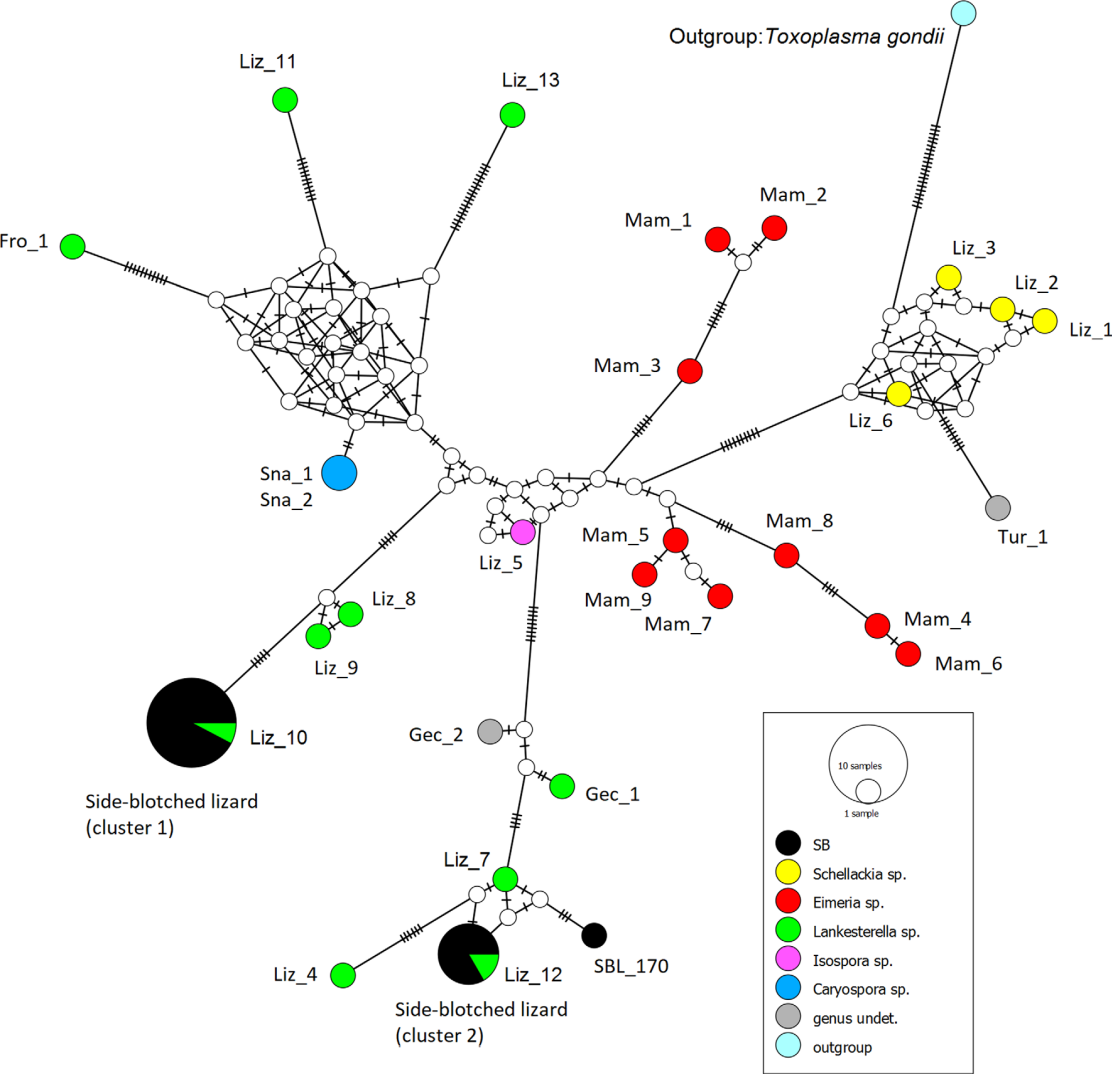
Median-joining haplotype network of parasite DNA sequences (18S rRNA gene). Details of the reference sequences, including blood parasite and host species identity and code and location, are given in Table 1. The size of the color-coded circles is proportional to haplotype frequency. The hatch marks represent mutational steps.

The median-joining haplotype network analysis (Figure 2) detected the same clusters and associations. The minimum distance between sequences of cluster 1 and cluster 2 (Figure 1) was 29 mutations (Figure 2). The network analysis further detected multiple nodes corresponding to hypothetical haplotypes not sampled.

### Microscopic examination

**Figure 3.**
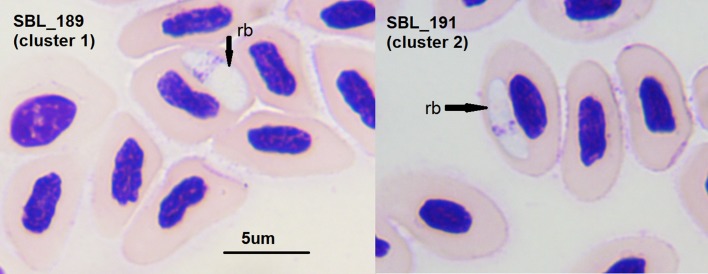
Two examples of erythrocytes from the side-blotched Lizards *Uta stansburiana* on San Benito Oeste Island infected with the blood parasite (sporozoite) detected in this study. Blood smears are stained with Giemsa.

Intraerythrocytic sporozoites (Figure 3): A single large sporozoite was seen in each erythrocyte. Sporozoites were elongated, convex on one side, and straight on the side next to the nucleus, with rather pointed ends. Some degree of hypertrophy of the host cell was variably noted, and only slight displacement of the host cell nucleus. Further characteristics were: pale cytoplasm (pale blue with Giemsa stain), nucleus in the form of a band of chromatin granules at one side of the center of the parasite, and a reserve vacuole (refractile body) was present, which was stained very pale orange.

## Discussion

In the present study, we analyzed blood samples collected from side-blotched lizards *Uta stansburiana* from San Benito Oeste Island, off the Mexican Pacific coast. We found two different blood parasite sequence clusters, which were closest to certain published sequences of *Lankesterella* (Figures 1 and 2).

The Apicomplexa are grouped into four groups designed to be utilitarian rather than to reflect evolutionary history [1, 3]: the coccidians, the gregarines, the hemosporidians, and the piroplasmids. These groups as well as the taxa contained within are not based on phylogenetic relationships, but on characteristics such as their associated host and/or vector [5, 20], and which particular tissues they inhabit. Their evolutionary relationships and their taxonomy are presently unclear (e.g. [7]), and the current classification does not take modern molecular data into account [3]).

Molecular data can be used to resolve previously unknown classifications. For example, in the present dataset, the samples Gec_2 and Tur_1 can be determined to genus level (*Lankesterella* sp. and *Schellackia* sp., respectively), based on Bayesian analysis (Figure 1).

Few studies have been published characterizing apicomplexan parasites in lizards at the molecular level, and the relationships of many of these protozoan species are unresolved, indicating that more work is required [19, 36]. This may explain some poor support values within the phylogenetic tree (Figure 1) and the occurrence of multiple nodes corresponding to hypothetical haplotypes not sampled in the network analyses (Figure 2). The sequence for *Lankesterella minima* (Fro_1) also illustrates the uncertainty: it was a sister group to all other *Lankesterella* in the Bayesian analysis (Figure 1), while grouping with both *Lankesterella* and *Caryospora* in the Maximum Likelihood analysis (Supplement 1) and the network (Figure 2). Morphologically, *Lankesterella minima* differed from the *Lankesterella* parasites observed here by having eosinophilic globules on each side of the nucleus [24]. Genetically, *Lankesterella minima* was also found to be closely related to *Caryospora* sequences in a study of blood parasites in sedge warblers *Acrocephalus schoenobaenus* (Aves [7]).

Another problem is the scarcity of differential phenotypic traits, which qualifies molecular phylogenetics based on genetic data as the best method to shed more light on the phylogenetic relationships among the coccidia [36].

The family Lankesterellidae belongs to the coccidians and is characterized by the fact that both the merogony and sporogony occur in the liver and intestine of the vertebrate. For this family the vertebrate thus acts as the definitive host. This means that no further development takes place in blood-sucking arthropods, especially mites, but also mosquitoes and sandflies [23], which take up the pathogens in the form of intra-erythrocytic sporozoites, and consequently act as purely mechanical carriers. As a consequence, host specificity is considered to be low on the side of the vector and infected vertebrate animals can also serve as a source of infection for other vertebrate animals [41, 45]. Experiments of transferring species of the genus *Schellackia* (Lankesterellidae) to new hosts by feeding infested mosquitoes and ticks failed [9, 21, 23]. On the side of the vertebrate hosts, in which the complete development takes place, however, specificity is high [33].

None of the sequences found in this study was 100% identical to previously published sequences and the genetic variability among the sequences found was relatively high. The lowest similarity observed between two sequences was 89% (Table 2) and the average difference between sequences of cluster 1 and cluster 2 was 4%. This difference is high compared to intraspecific differences in coccidians in other studies. For example, isosporoid coccidia (*Isospora* and *Atoxoplasma* spp.) in most passerine birds had average distances of 0.1% (i.e. 99.9% similarities [42]), but some exceptions were also observed (3.5% between genotypes in cowbirds *Molothrus ater* [42]). Most likely, a 4% difference would indicate that cluster 1 and cluster 2 sequences belong to different species of *Lankesterella.*

However, criteria for the differentiation of *Lankesterella* species are limited (e.g. the sporozoite shape) and a system for taxonomic differentiation has not been developed. Sporozoites are mostly described in peripheral blood erythrocytes, while other stages of the life cycle, e.g. the liver or intestine stages may be informative. Moreover, since the sporozoites lack specific micro-morphological differences, light microscopy of blood smears is not suitable for identification to the species level [46]. Electron microscopic fine structure analyses would be needed to determine specific micro-morphological differences.

In conclusion, in the present study we found new haplotypes of *Lankesterella sp.* infecting side-blotched lizards on a remote oceanic island of the Pacific Ocean off Mexico. We also found high genetic variability, including genetic similarities (i.e. related haplotypes with maximum 99% similarity) and differences (new haplotypes) between the blood parasites of the island population and the population further north on the mainland. Given the wide distribution range of this species on different islands and on the continent, this species would present a good study model for the microevolution of lizard and parasitic haplotypes.

## Conflict of Interest

We declare that we have no competing interests.
